# Platinum Nanoparticles Loaded in Polydopamine-Modified Porous Coordination Network-224 with Peroxidase-Like Activity for Sensitive Glutathione Detection

**DOI:** 10.3390/biom15071002

**Published:** 2025-07-13

**Authors:** Shoubei Gao, Mingyue Gao, Chenran Zhen, Yanshuai Cui, Xianbing Ji, Ruyu Li, Longgang Wang

**Affiliations:** 1 Hebei Key Laboratory of Agroecological Safety, Department of Environmental Engineering, Hebei University of Environmental Engineering, Qinhuangdao 066102, China; shoubei@stumail.ysu.edu.cn (S.G.); jixianbing@hebuee.edu.cn (X.J.); 2 State Key Laboratory of Metastable Materials Science and Technology, Hebei Key Laboratory of Nano-Biotechnology, Hebei Key Laboratory of Applied Chemistry, Yanshan University, Qinhuangdao 066004, China; gmy1999@stumail.ysu.edu.cn (M.G.); zcran@stumail.ysu.edu.cn (C.Z.); liruyuys@stumail.ysu.edu.cn (R.L.)

**Keywords:** PCN-224, nanozyme, glutathione, colorimetric detection

## Abstract

The content of glutathione in the human body is crucial to human health, so a convenient and efficient method is needed to detect it. Herein, porous coordination network-224 (PCN-224) was modified by polydopamine to prepare polydopamine-modified PCN-224 (PCN-224-PDA) to improve the water dispersibility of the PCN-224. Monodispersed platinum nanoparticles were loaded into the PCN-224-PDA to prepare PCN-224-PDA-Pt. The PCN-224-PDA-Pt showed high peroxidase-like catalytic activity, and its catalytic activity was affected by pH and temperature. The PCN-224-PDA-Pt almost had no hemolysis of red blood cells. In addition, the PCN-224-PDA-Pt showed high affinity for 3,3′,5,5′-tetramethylbenzidine and catalytic efficiency in kinetic studies, and the type of reactive oxygen species generated during the catalytic process was hydroxyl radicals. More importantly, a colorimetric method for glutathione detection was developed based on the peroxidase-like activity of the PCN-224-PDA-Pt. The linear detection range was 1–600 μM and the detection limit reached 0.306 μM. This method shows good anti-interference capabilities and excellent recovery rates, indicating its strong potential for applications in biological detection.

## 1. Introduction

Glutathione (GSH) is a critical non-protein thiol compound within cells. GSH is primarily responsible for regulating redox balance, neutralizing free radicals, and maintaining normal cellular functions [1]. GSH concentrations in healthy individuals are 1–10 μM in the plasma and 5–10 mM in the liver. These levels shift markedly in disease: cancers show increased GSH (e.g., 8–12 mM in liver tumors), while neurodegeneration exhibits reduced GSH (e.g., 1.5–2.1 mM in the Alzheimer’s brain). These changes are directly linked to the pathogenic oxidative stress mechanisms. Therefore, developing efficient and sensitive methods for GSH detection is crucial for the early diagnosis and monitoring of diseases [2,3].

Among the various detection methods, colorimetric analysis has gained widespread attention due to its convenience and simplicity [4,5]. In colorimetric detection methods, natural enzymes are used in GSH detection. Artificial enzymes can mimic the catalytic performance of natural enzymes [6]. The Yan group discovered iron (II, III) oxide (Fe_3_O_4_) nanoparticles with peroxidase-like activity in 2007 [7]. Subsequently, a variety of artificial enzymes have been explored, especially precious metal nanozymes such as Pt [8], Pd [9], and Au [10], which have shown tremendous potential application in biosensing due to their outstanding catalytic activity [11,12]. Compared to natural enzymes, these precious nanozymes have large-scale production and tuning performance [13]. However, their tendency to aggregate can lead to reduced specific surface areas and diminished catalytic performance [14].

Metal–organic frameworks (MOFs) are a good platform for the construction of nanozymes. MOFs have highly ordered porous structures, tunable chemical functionalities, and excellent surface areas [15]. Porous coordination network-224 (PCN-224) is composed of unique Zr_6_ clusters and porphyrin tetracarboxylate ligands [16,17]. PCN-224 can serve as a carrier for noble metal nanoparticles, such as Pt@PCN-224 [18], Ag@PCN-224 [19], and Au@PCN-224 [20]. However, these nanozymes still lack stability in their solutions. To further enhance the dispersibility of PCN-224 in aqueous solutions, PCN-224 has been functionalized by hyaluronic acid and polydopamine (PDA) [21,22]. Dopamine (DA) has the excellent biocompatibility and self-polymerization properties. Polydopamine (PDA) ensures the good stability and dispersibility of PCN-224 [23,24]. Therefore, utilizing polydopamine-modified PCN-224 (PCN-224-PDA) as a carrier of noble nanoparticles holds excellent research prospects for addressing the issue of the easy aggregation of dispersed noble metal nanoparticles.

Herein, PCN-224-PDA was used as a template to load monodispersed Pt nanoparticles to prepare polydopamine-modified, PCN-224-loaded Pt nanoparticles (PCN-224-PDA-Pt). The PCN-224-PDA-Pt displayed excellent peroxidase-like performance, improved dispersion, and enhanced stability. This research enhances the dispersion of PCN-224 in aqueous solutions and prevents the aggregation of precious metal nanozymes.

## 2. Materials and Methods

### 2.1. Materials

Zirconium oxychloride octahydrate (ZrOCl_2_·8H_2_O), benzoic acid, potassium tetrachloroplatinate (II) (K_2_PtCl_4_), tris (hydroxymethyl) aminomethane hydrochloride (Tris-HCl), sodium borohydride (NaBH_4_), 3,3′,5,5′-tetramethylbenzidine (TMB), o-phenylenediamine (OPD), 30% hydrogen peroxide (30% H_2_O_2_), dopamine hydrochloride (DA·HCl), glutathione (GSH), and terephthalic acid (TA) were bought from Aladdin Reagent Co., Ltd. (Shanghai, China) Tetra (4-carboxyphenyl) porphyrin (TCPP) was bought from Shanghai Bide Pharmatech Co., Ltd. (Shanghai, China) N,N-dimethylformamide (DMF) was bought from Tianjin Kemio Chemical Reagent Co., Ltd. (Tianjin, China); and 2,2′-azinobis (3-ethylbenzothiazoline-6-sulfonic acid) diammonium salt (ABTS) was bought from Shanghai Ruji Biotechnology Development Co., Ltd. (Shanghai, China).

### 2.2. Preparation of Polydopamine-Modified PCN-224 Loaded Pt Nanoparticles

PCN-224 was prepared by the water bath method. ZrOCl_2_·8H_2_O (240 mg), TCPP (80 mg), and benzoic acid (2.24 g) were dissolved in 80 mL of DMF. After ultrasonication for 10 min, the mixture was placed in a water bath at 90 °C. After the temperature of the reaction system was cooled to room temperature, PCN-224 particles were obtained by centrifugal separation (9000 rpm, 10 min). The prepared PCN-224 particles were centrifuged and sequentially washed three times with DMF, followed by three washes with ultrapure water. Finally, the PCN-224 particles were vacuum-dried to obtain a solid purple powder, which was protected from light.

Totals of 20 mg of PCN-224 solid powder and 8 mg of dopamine hydrochloride were dissolved in a Tris-HCl buffer solution with a pH of 8.5. This mixture was stirred at room temperature for 12 h in the dark. The product, PCN-224-PDA, was then collected by centrifugation and washing. Subsequently, the PCN-224-PDA was vacuum-dried to obtain a solid black powder, which was protected from light.

The prepared PCN-224-PDA was completely dispersed in deionized water to obtain a 2 mg/mL aqueous suspension. A total of 250 μL of this solution was taken, and 100, 200, and 300 μL of 10 mM K_2_PtCl_4_ were added, respectively. An appropriate amount of water was added to adjust the solution volume to 1450 μL. The pH was adjusted to about 4 using 1 M hydrochloric acid. The mixture was stirred for 2 h. Then, 50 μL of 0.5 M NaBH_4_ was added. The final volume was adjusted to 1500 μL. After stirring for 20 min, the pH was adjusted to neutral. The stirring continued for 12 more hours, and then the product was dialyzed for 8 h. The water was changed three times to obtain polydopamine-modified PCN-224 loaded Pt nanoparticles (PCN-224-PDA-Pt).

### 2.3. Characterization of Catalytic Activity of PCN-224-PDA-Pt

In the peroxidase-like activity assay experiment, 50 μL of PCN-224-PDA-Pt was added to a 2 mL centrifuge tube, followed by the addition of 200 μL of 0.2 M acetic acid–sodium acetate buffer solution (pH 4), 1000 μL of 0.6 mM TMB solution, and 100 μL of 0.3 M H_2_O_2_ solution. The sample was incubated for 5 min at 25 °C and 600 rpm in a constant-temperature mixer. After incubation, the absorbance was measured by a UV-vis spectrometer to evaluate the peroxidase-like activity of the catalyst.

To investigate the optimal pH conditions, 50 μL of PCN-224-PDA-Pt was added to 2 mL centrifuge tubes. Then, 200 μL of acetic acid–sodium acetate buffer solutions covering a pH range from 2 to 12 were added. Next, 1000 μL of TMB solution (0.6 mM) prepared in the corresponding pH buffers and 100 μL of H_2_O_2_ (0.3 M) were added. The samples were incubated at 25 °C and 600 rpm for 5 min, and the absorbance at 652 nm was measured using a UV-vis spectrometer to evaluate the peroxidase-like activity.

For the optimal temperature study, the samples were then incubated at temperatures ranging from 10 °C to 70 °C at 600 rpm for 5 min. The absorbance at 652 nm was measured using a UV-vis spectrometer to evaluate the peroxidase-like activity at different temperatures.

### 2.4. Hemolysis Experiment

Red blood cells were diluted with physiological saline to prepare a 2% *v*/*v* red blood cell suspension. Sample solutions were prepared with concentrations ranging from 1.56 μg/mL to 200 μg/mL. An equal volume of each sample solution was mixed with the red blood cell suspension and incubated. The supernatant was transferred into a 96-well plate.

The absorbance of the supernatants at 575 nm was measured using a microplate reader. Physiological saline containing 2% *v*/*v* red blood cell suspension was used as the negative control, and deionized water containing 2% *v*/*v* red blood cell suspension was used as the positive control. The hemolysis rate was calculated from Formula (1), where OD_sample_, OD_positive_, and OD_negative_ represented the absorbances at 575 nm of the sample, positive control, and negative control, respectively.

(1) $$Hemolysis\;rate\left(\%\right)=\frac{{OD}_{sample}-{OD}_{negative}}{{OD}_{positive}-{OD}_{negative}}\times 100\%$$

### 2.5. Study of Catalytic Kinetics

In the catalytic kinetic study of peroxidase-like activity focusing on TMB concentration, 50 μL of PCN-224-PDA-Pt was first added to each 2 mL centrifuge tube. Acetic acid–sodium acetate buffer solution (pH = 4, 0.2 M) was then added to the tubes, with volumes ranging from 1100 μL to 100 μL, decreasing by 100 μL each time. Correspondingly, TMB (pH = 4, 0.6 mM) was added in volumes ranging from 100 μL to 1100 μL, increasing by 100 μL each time. To maintain the total liquid volume in the centrifuge tube at 1350 μL, an additional 100 μL of 0.3 M H_2_O_2_ was added. The changes in sample absorbance over time were measured using a UV-visible spectrophotometer at a wavelength of 652 nm to further study the enzyme catalytic kinetics. The catalytic kinetic data of H_2_O_2_ were obtained by a similar method.

### 2.6. Study on the Mechanism of Peroxidase-Like Activity

Terephthalic acid (TA) was used as the probe to detect hydroxyl radicals (•OH) generated by PCN-224-PDA-Pt exhibiting peroxidase-like activity. A mixture was prepared by adding 50 μL of PCN-224-PDA-Pt, 1000 μL of TA solution (0.5 mM), 100 μL of H_2_O_2_ (0.3 M), and 200 μL of acetic acid–sodium acetate buffer (pH = 4, 0.2 M). This mixture was then incubated for 2 h with shaking at 25 °C and 600 rpm using a thermostatic mixer to perform the hydroxyl radical capture experiment according to reference [25].

### 2.7. Glutathione Determination

A total of 50 μL of PCN-224-PDA-Pt was added into a 2 mL centrifuge tube, followed by the addition of 1000 μL of TMB (pH = 4, 0.6 mM) and 100 μL of H_2_O_2_ (0.3 M). The mixture was reacted in a thermostatic mixer at 25 °C and 600 rpm for 5 min. Subsequently, 200 μL of glutathione aqueous solutions at different concentrations were added and the reaction was continued for an additional 3 min. The absorbance was then measured using a UV-vis spectrophotometer. The relationship between the absorbance difference and concentration was used to create a standard curve for detection, with the glutathione concentration range being 0–5 mM. The actual samples for recovery rate testing were serum and cheat flour.

Different interfering substances, namely histidine, phenylalanine, alanine, leucine, lysine, proline, tyrosine, glycine, Mg^2+^, K^+^, Ca^2+^, and Na^+^, were added into the test tube. The absorbance was measured to determine the anti-interference ability of the nanozymes. During the experiment, the detection substance was replaced with an interfering substance, and the concentration of the interfering substance was set at 6 mM.

## 3. Results and Discussion

### 3.1. Structure Analysis of PCN-224-PDA-Pt

PCN-224 is a metal–organic framework material formed by the self-assembly reaction of tetravalent zirconium ions with a tetracarboxylic porphyrin ligand, where all four end groups are carboxylic acids [26]. We utilized the self-polymerization properties of dopamine hydrochloride in an alkaline environment to form polydopamine on PCN-224 to enhance its dispersibility in aqueous solutions [27]. This resulted in the formation of PCN-224-PDA, onto which Pt nanoparticles were subsequently loaded.

**Figure 1:**
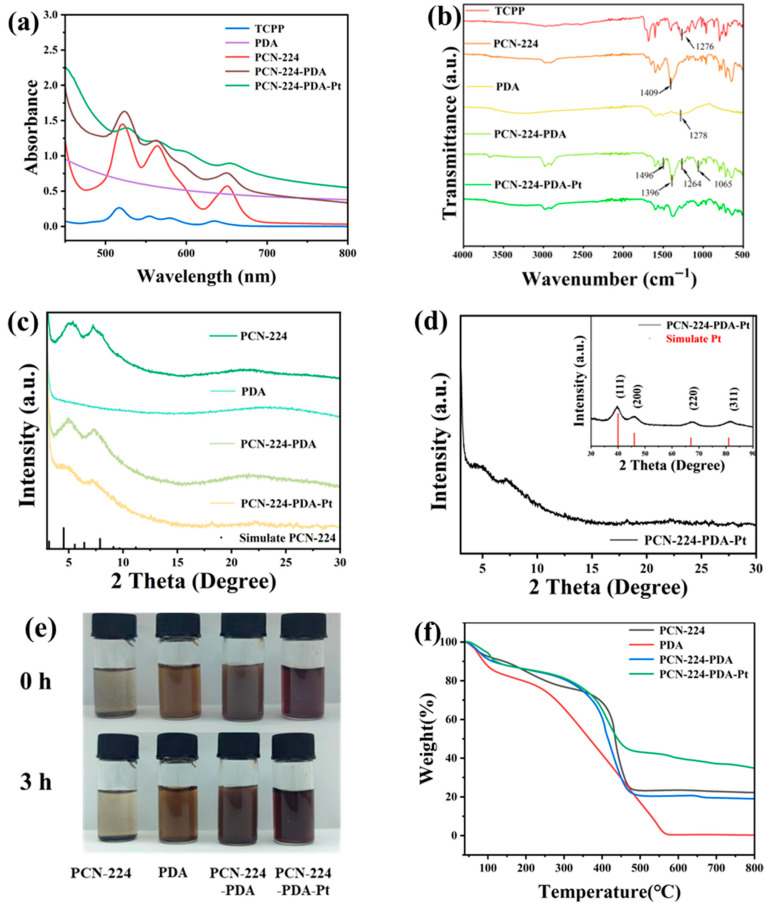
(**a**) UV-vis spectra of different samples, (**b**) infrared spectra of different samples, (**c**) XRD spectra of different samples, (**d**) XRD spectrum of PCN-224-PDA-Pt, (**e**) optical characterization of different substances in water, and (**f**) thermogravimetric analysis of PCN-224-PDA-Pt.

The UV-vis spectra of a series of materials are shown in Figure 1a. After the combination of Zr^4+^ and TCPP, the Q-band absorption peak of TCPP is consistent with that of PCN-224, indicating the successful preparation of PCN-224 [28]. The polydopamine (PDA) does not show characteristic peaks in this range, and the absorbance gradually decreases with increasing wavelengths. It can be observed that the overall absorbance of the PCN-224-PDA increases at the same concentration after modifying with polydopamine on the exterior. The absorbance of the PCN-224-PDA-Pt further increases after the loading of the precious metal platinum Pt on its surface. However, the peak variations at ~650 nm in the Q-band region decrease from PCN-224 to PCN-224-PDA-Pt.

As shown in of Figure 1b, the characteristic bands of the TCPP at 1650 cm^−1^ (C=O) and 1276 cm^−1^ (C-OH) show significantly reduced transmittance rates in the PCN-224, indicating coordination of the carboxyl groups with Zr^4+^ ions, confirming the successful synthesis of PCN-224 [29]. A strong peak at 1409 cm^−1^ in the PCN-224 spectrum corresponds to the COO- peak [30]. In the spectrum of the PCN-224-PDA, this peak shifts to 1396 cm^−1^, which is caused by the formation of hydrogen bonds between the COO- groups in the PCN-224 and the numerous amino groups in the PDA. In the PCN-224-PDA spectrum, two sharp peaks at 1278 cm^−1^ and 1500 cm^−1^ originate from the bending and stretching vibrations of C-O-H. Additionally, two strong peaks at 1496 cm^−1^ and 1065 cm^−1^ correspond to the vibration peaks of N-H and C-O, respectively, confirming the presence of dopamine in the particles [30,31]. These results demonstrate that the PCN-224 is indeed modified with a PDA layer in the PCN-224-PDA.

X-ray diffraction was used to investigate the structural changes in the PCN-224-PDA-Pt. As shown in Figure 1c, the crystal structure of the spherical PCN-224 closely corresponds to the simulated standard structure, indicating the successful synthesis of the PCN-224. The reference for the simulated PCN-224 is derived from the CIF file of Dawei Feng et al. [17]. The intensity of the diffraction peaks of the PCN-224-PDA gradually decreases, yet the basic structure remains unchanged. As observed from Figure 1d, the crystal planes of the PCN-224-PDA-Pt at (111), (200), (220), (311), and (222) correspond to diffraction angles of 39.87°, 46.26°, 67.68°, 81.43°, and 86.12° after the introduction of Pt nanoparticles, respectively [32]. Compared with the reference code 01-001-1194 of the metal Pt standard card, the Pt nanoparticles loaded on the surface of the PCN-224-PDA exhibit a face-centered cubic crystal structure. Thus, we further confirmed the successful synthesis of the PCN-224-PDA-Pt by measuring its X-ray diffraction angles and determined that the crystal structure of the Pt was face-centered cubic.

As observed from Figure 1e, the PCN-224, PDA, PCN-224-PDA, and PCN-224-PDA-Pt were dispersed in water. The PCN-224 solution has formed noticeable particle sedimentation at the bottom after 3 h, and the supernatant has become clear. In contrast, the PCN-224-PDA solution has remained uniform overall, with no evident sedimentation. Similarly, the PCN-224-PDA-Pt solutions show no significant changes and remain as uniform liquids. These observations indicate that the water dispersibility of PCN-224-PDA is significantly better than that of PCN-224, likely due to the strong hydrophilicity of PDA. Furthermore, the PCN-224-PDA-Pt has also maintained good dispersion in water after Pt loading. This reinforces the important role of the PDA outer layer in PCN-224-PDA, particularly in enhancing the stability and dispersion of the material in water.

Thermal stability analysis of the prepared PCN-224, PCN-224-PDA, PDA, and PCN-224-PDA-Pt was conducted using a thermogravimetric analyzer. The weight loss for the PDA also occurred in three stages: the initial stage, from 40 to 100 °C, involved the removal of moisture; the second stage, from 100 to 250 °C, was characterized by the breakdown of unstable structures; and the third stage, from 250 to 550 °C, involved the decomposition and disintegration of the entire structure.

Conversely, the weight loss process for the PCN-224, PCN-224-PDA, and PCN-224-PDA-Pt is divided into three stages in Figure 1f. Initially, the weight loss primarily results from the removal of water adsorbed within the structure in the temperature range of 40–100 °C. The loss is attributed to the removal of organic solvents adsorbed within the structure in the temperature range of 100–350 °C [33,34]. The third stage, occurring between 350 and 475 °C, involves weight loss due to the decomposition of benzene and porphyrin rings in the organic ligands and the collapse of the overall MOF structure [35]. Finally, the residual weights of the PCN-224, PCN-224-PDA, PDA, and PCN-224-PDA-Pt were approximately 22.27%, 19.05%, 0.26%, and 34.88%, respectively.

**Figure 2:**
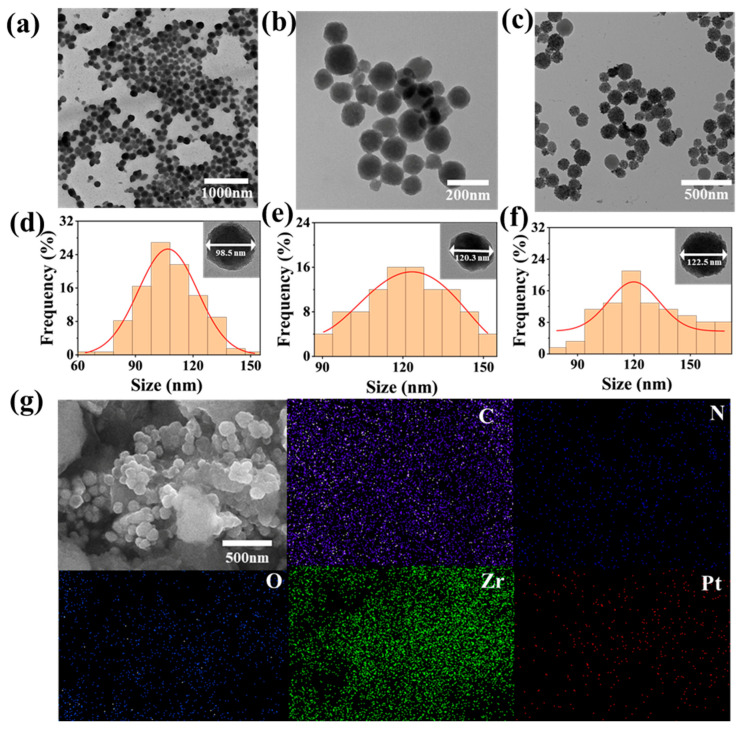
TEM images and particle size statistics: (**a**,**d**) PCN-224, (**b**,**e**) PCN-224-PDA, and (**c**,**f**) PCN-224-PDA-Pt; (**g**) SEM image and different element distributions of PCN-224-PDA-Pt.

The basic morphologies of the materials were observed using transmission electron microscopy (TEM). As shown in Figure 2a,d, uniform spherical PCN-224 nanoparticles have successfully been synthesized, with a particle size of predominantly 98.2 ± 14.4 nm. As is visible in Figure 2b,e, the PCN-224-PDA exhibits an irregular spherical shape and a rough surface texture after modifying of the surface with a layer of PDA. The size of the PCN-224-PDA has increased to an approximate 121.7 ± 16.5 nm. Further functionalization with platinum nanoparticles (Pt NPs) was evident in Figure 2e,f, where the Pt NPs are visibly dispersed on the surface of the PCN-224-PDA-Pt. Notably, the overall particle size of the PCN-224-PDA-Pt (127.2 ± 23.0 nm) showed no significant change compared to the PCN-224-PDA, suggesting that the Pt deposition did not alter the core morphology. In Figure 2g, the morphology of the composite PCN-224-PDA-Pt is observed to be roughly spherical, and the particle size is consistent with that shown in Figure 2c. The elements C, N, O, Zr, and Pt within the composite material PCN-224-PDA-Pt are evenly distributed. The elemental analysis of the total spectrum confirmed the successful synthesis of the PCN-224-PDA-Pt.

**Figure 3:**
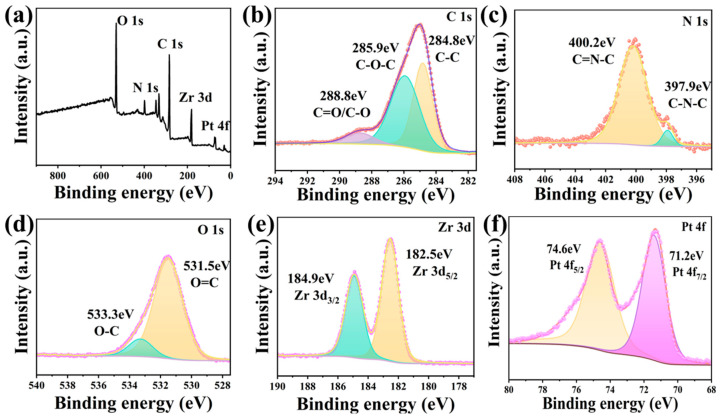
XPS characterization of PCN-224-PDA-Pt: (**a**) full spectrum, (**b**) C 1s, (**c**) N 1s, (**d**) O 1s, (**e**) Zr 3d, and (**f**) Pt 4f.

X-ray photoelectron spectroscopy (XPS) was used to further analyze the surface element composition, chemical state, and molecular structure of the PCN-224-PDA-Pt. The PCN-224-PDA-Pt displays elemental signals for C, N, O, Zr, and Pt in the full spectrum in Figure 3a, confirming that the Pt is successfully coordinated within the PCN-224-PDA. The C 1s spectrum in Figure 3b exhibits absorption peaks at 288.8 eV, 286.3 eV, and 284.8 eV, attributed to aromatic C=O/C-O, C-N, and C-C, respectively [36]. The N 1s spectrum in Figure 3c appears at 400.2 eV and 397.9 eV, corresponding to C=N-C and C-N-C, respectively. The O 1s spectrum in Figure 3d shows peaks at 533.3 eV and 531.5 eV, attributed to O-H and C=O, respectively [6]. The Zr 3d spectrum in Figure 3e displays two characteristic peaks, Zr 3d_3/2_(185.0 eV) and Zr 3d_5/2_ (182.5 eV), indicating the presence of Zr (IV) in the structure [37]. The Pt 4f spectrum in Figure 3f reveals peaks at 74.6 eV and 71.2 eV, corresponding to Pt 4f_5/2_ and Pt 4f_7/2_, respectively, indicating that the Pt is in the zero-valent state in the PCN-224-PDA-Pt.

### 3.2. Enzyme-Like Activity Analysis of PCN-224-PDA-Pt

**Figure 4:**
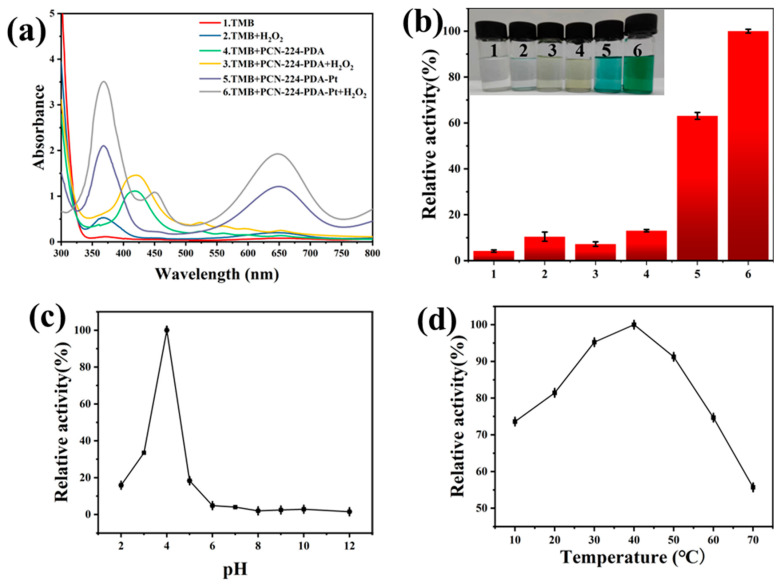
Characterization of enzyme-like activity: (**a**) UV-vis spectra of different groups; (**b**) relative activity of Figure (**a**) at 652 nm, with optimal reaction conditions for the relative peroxidase-like activity of PCN-224-PDA-Pt; (**c**) different pH values; and (**d**) different temperatures.

TMB was selected as the reaction substrate in exploring the enzyme-like properties of the PCN-224-PDA-Pt. As shown in Figure 4a,b, groups 1, 2, 3, and 4 have no obvious absorbance changes or color changes at 652 nm. The solution of group 5 [TMB+PCN-224-PDA-Pt] has turned light blue in the absence of H_2_O_2_, which indicates that PCN-224-PDA-Pt exhibits oxidase-like activity. Furthermore, the solution of group 6 [TMB+PCN-224-PDA-Pt+H_2_O_2_] has turned blue green in the presence of H_2_O_2_, which indicates that PCN-224-PDA-Pt exhibits peroxidase-like activity. The absorbance of group 6 is much higher than that of group 5. Consequently, the peroxidase-like activity of PCN-224-PDA-Pt was selected for further investigation in subsequent studies.

Next; we selected different chromogenic substrates, OPD and ABTS, to further verify the catalyst’s activity. As shown in Figure S2, PCN-224-PDA-Pt catalyzes the color changes in these three chromogenic substrates with the appearance of characteristic peaks in the presence of H_2_O_2_. The colorless OPD is oxidized to yellow 2,3-diaminophenazine with a characteristic absorption peak at 450 nm by the PCN-224-PDA-Pt. The ABTS has turned green and exhibits a characteristic absorption peak at 420 nm after catalyzation by the PCN-224-PDA-Pt. This demonstrates that PCN-224-PDA-Pt can react with a variety of chromogenic substrates and possesses robust and efficient peroxidase-like properties.

We further examined the catalytic activity of the PCN-224-PDA-Pt under varying pH values and temperatures. Figure 4c shows that the PCN-224-PDA-Pt has achieved optimal catalytic performance at pH 4. When the pH exceeds 5, its catalytic activity sharply decreases and nearly ceases, likely due to the instability of the hydrogen peroxide at high pH levels. Additionally, temperature significantly impacts the activity of nanozymes. As depicted in Figure 4d, the PCN-224-PDA-Pt maintains over 50% of its catalytic activity within a temperature range of 20 °C to 70 °C. The activity curve exhibits a pattern of initially increasing and then decreasing with rising temperatures. The optimal catalytic activity occurs at 40 °C, which is identified as the best temperature for this catalyst to perform catalytic reactions. Overall, the most favorable conditions for the catalytic reaction of the PCN-224-PDA-Pt nanozyme are at 40 °C and a pH of 4.

### 3.3. Hemolysis Analysis of PCN-224-PDA-Pt

In the hemolysis experiment, we evaluated the impact of PCN-224-PDA-Pt on human cells from volunteers’ blood. That experiment used physiological saline as a negative control and deionized water as a positive control. Different concentrations of PCN-224-PDA-Pt were dissolved in the physiological saline, and that solution was then mixed with a red-blood-cell suspension. After incubation, the hemolysis rate was determined by centrifuging the mixture to separate the supernatant and measuring its absorbance. As shown in Figure S3, the hemolysis rate of the PCN-224-PDA-Pt remains below 5% within the selected concentration range. The nearly colorless appearance of the supernatant indicates that PCN-224-PDA-Pt is highly compatible with red blood cells and has minimal impact on human cells, demonstrating its strong potential for biomedical applications.

### 3.4. Analysis of the Catalytic Kinetics and Peroxidase-Like Activity Mechanism of PCN-224-PDA-Pt

To further study the catalytic kinetic properties of PCN-224-PDA-Pt, two key sets of experiments were designed: one to observe the impact on the catalytic reaction by varying the concentration of TMB, and the other to adjust the concentration of hydrogen peroxide to analyze its effect on reaction kinetics. These experimental approaches aimed to gain a deeper understanding of how the catalyst affects reaction rates. In the study of enzyme-catalyzed reactions, the Michaelis constant (*K_m_*) is an indicator that reflects the affinity between an enzyme and a substrate. A smaller *K_m_* value indicates a higher affinity. The maximum reaction rate (*V_max_*) reflects the catalytic efficiency and the maximum catalytic capacity of the enzyme.

**Figure 5:**
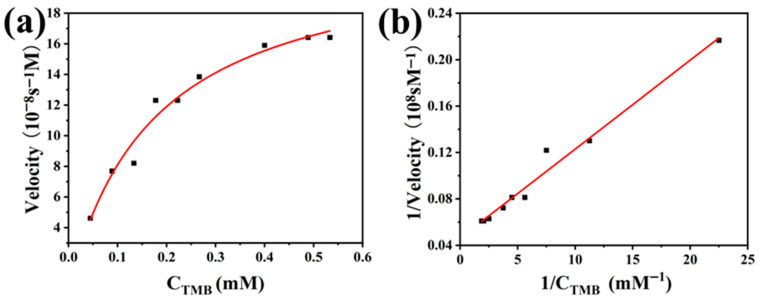

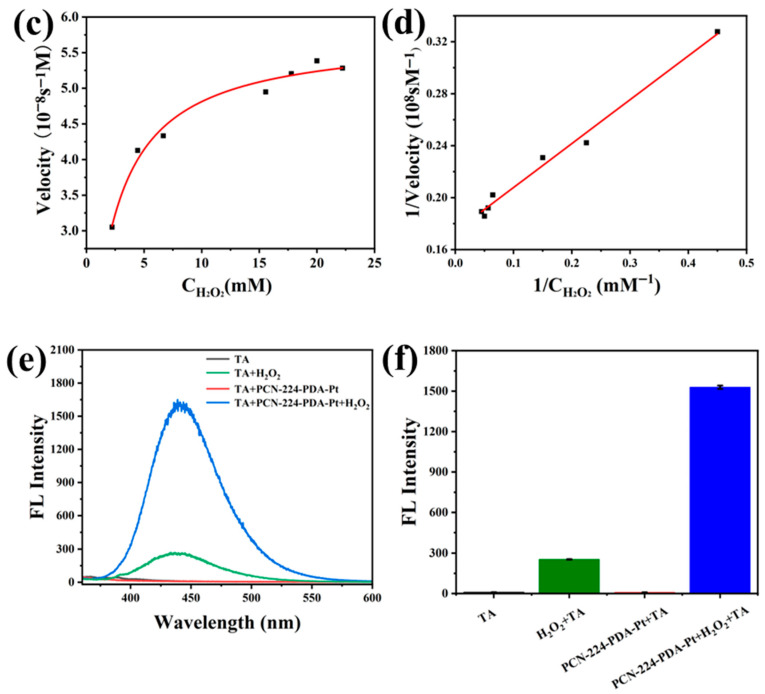
Characterization of catalytic kinetics and peroxidase-like activity mechanism of PCN-224-PDA-Pt: (**a**) catalytic kinetics diagram with different TMB concentrations; (**b**) double reciprocal curve of (**a**); (**c**) catalytic kinetics diagram with different H_2_O_2_ concentrations, (**d**) double reciprocal curve of (**c**); mechanism of peroxidase-like activity of PCN-224-PDA-Pt; (**e**) fluorescence emission spectra of different reaction groups; and (**f**) relative activity of (**e**) at 435 nm.

As shown in Figure 5a, the reaction rate exhibits a noticeable trend corresponding to the changes in the TMB concentration. This behavior aligns with the Michaelis–Menten kinetic model. Through the double reciprocal plot in Figure 5b, we calculated the *K_m_* for the substrate TMB to be 0.177 mM and *V_max_* to be 2.216 × 10^−7^ Ms^−1^. Similarly, as depicted in Figure 5d, *K_m_* was 1.940 mM and *V_max_* was 5.747 × 10^−8^ Ms^−1^ for the substrate hydrogen peroxide. The higher *K_m_* value for the hydrogen peroxide compared to the TMB indicates that PCN-224-PDA-Pt has a lower affinity for hydrogen peroxide. Additionally, the lower *V_max_* for hydrogen peroxide, when compared to TMB, suggests that PCN-224-PDA-Pt is less efficient in catalyzing reactions involving hydrogen peroxide than those involving TMB.

To verify whether the peroxidase-like PCN-224-PDA-Pt would generate hydroxyl radicals (•OH) during the catalytic reaction, we used terephthalic acid (TA) as a quencher in the experiment. TA reacts with hydroxyl radicals to produce 2-hydroxyterephthalic acid (HTA), which emits fluorescence at a wavelength of 435 nm under 415 nm light excitation. As shown in Figure 5e, the group of [TA+H_2_O_2_+PCN-224-PDA-Pt] exhibits the strongest fluorescence intensity, at 435 nm, indicating that hydroxyl radicals are indeed generated during the catalytic process. The control group, containing only [H_2_O_2_+TA], also shows some fluorescence intensity, possibly due to the decomposition of H_2_O_2_ itself producing a small amount of hydroxyl radicals. In contrast, the control groups with only TA and [TA+PCN-224-PDA-Pt] show almost no fluorescence intensity, indicating that PCN-224-PDA-Pt alone cannot generate hydroxyl radicals without the presence of H_2_O_2_. Figure 5f displays the fluorescence intensities at 435 nm for the four experimental groups. The fluorescence intensity of the group [H_2_O_2_+TA] was 253, while the fluorescence intensity of the group [TA+H_2_O_2_+PCN-224-PDA-Pt] reached 1529. This indicates the formation of ·OH during the catalytic process.

### 3.5. Glutathione Detection Analysis

**Figure 6:**
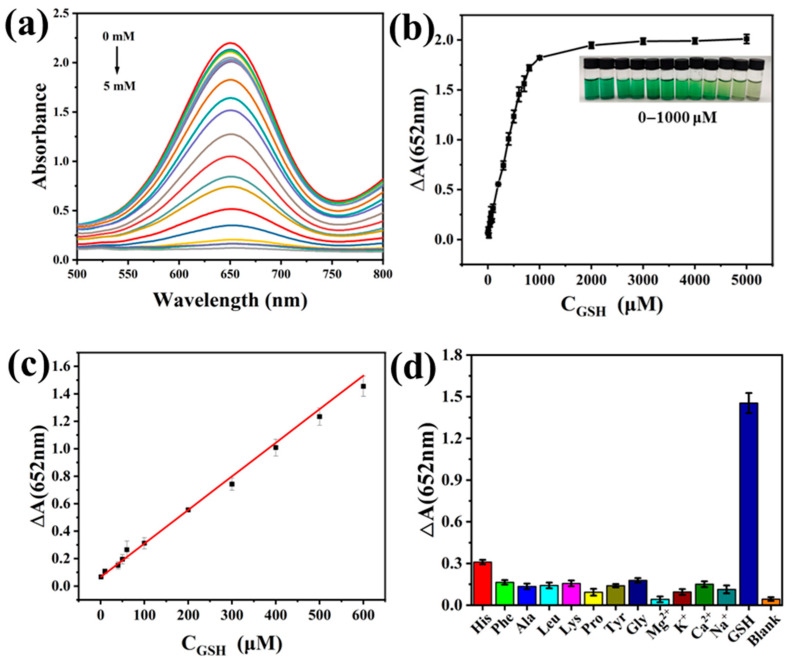
(**a**) UV-vis spectra for the detection of GSH, (**b**) GSH absorbance difference (ΔA) versus GSH concentration, (**c**) linear-fitting graph between ΔA and GSH in the range of 1–600 μM, and (**d**) effect of interfering agents on GSH detection.

GSH plays a crucial role as an antioxidant in organisms. It possesses reducing properties and can reduce a variety of substances. Notably, GSH can reduce oxTMB to colorless TMB. We developed a convenient method for detecting GSH. Different concentrations of GSH were added to the PCN-224-PDA-Pt+TMB+H_2_O_2_ system, and a UV-vis spectrometer was used to detect the absorption spectrum of the solution. As shown in Figure 6a, as the GSH concentration continues to increase, the color of the solution becomes lighter and the absorbance at 652 nm decreases. As shown in Figure 6b,c, the initial absorbance minus the subsequent absorbance after reduction is named as ΔA. As the GSH concentration increases, the curve exhibits a linear relationship in the range of 1–600 μM. The change in the absorbance difference is nearly constant beyond 1000 μM. Therefore, we selected the 1–600 μM range for linear fitting. The fitting equation is ΔA = 0.00245 × C_GSH_ + 0.06502 (R^2^ = 0.999) in Figure 6c. Thus, the detection range is 1–600 μM, and the detection limit is 0.306 μM. By comparing this to the different materials in Table 1, it could be found that PCN-224-PDA-Pt has a wider detection range and a lower detection limit.

In the anti-interference experiment, an interfering agent was used to replace the GSH and added to the PCN-224-PDA-Pt+TMB+H_2_O_2_+GSH system. In that experiment, the concentration of GSH was 600 μM and the concentration of the interfering agent was 6 mM. The interfering agents included histidine (His); phenylalanine (Phe); alanine (Ala); leucine (Leu); lysine (Lys); proline (Pro); tyrosine (Tyr); glycine (Gly); and ions such as Mg^2+^, K^+^, Ca^2+^, and Na^+^. As shown in Figure 6d, although the concentration of the interfering agents added was ten times that of the GSH, the interfering agents had little effect on the absorbance. Therefore, PCN-224-PDA-Pt exhibits good anti-interference performance as a probe for detecting GSH.

In addition, PCN-224-PDA-Pt was used to determine the recovery rate of the GSH in the sample. Serum and flour were taken as actual sample solutions, and the standard addition method was used to detect the recovery rate of the GSH in the actual sample solution using the standard curve. The recovery rate of the GSH was between 101.50% and 105.31% in the serum sample, and the relative standard deviation was less than 3%. The recovery rate of the GSH detection was between 101.59% and 103.26% in the flour solution, and the relative standard deviation was less than 3%. These test results show the reliability of the detection method.

## 4. Conclusions

In summary, we prepared PCN-224-PDA with good dispersion and used it as a carrier to load platinum nanoparticles. The PCN-224-PDA-Pt showed a good peroxidase-like catalytic effect. The type of reactive oxygen species generated during the catalytic process was •OH. In addition, we adopted a colorimetric method to detect GSH, and the detection range was 1–600 μM with a detection limit of 0.306 μM. The material also exhibited good anti-interference effects and excellent recovery rates.

## Figures and Tables

**Table 1:** Comparison of GSH detection ranges and detection limits of different materials.

Material	Linear Range (μM)	Detection Limit (μM)	Reference
PCN-224-PDA-Pt	1–600	0.306	This work
CoOOH	33–1300	13.70	[38]
MnO_2_	1–25	0.30	[39]
Au(III)/CDC	1–300	3.65	[40]
Ag NP	0–400	4.11	[41]
Fe_3_O_4_ NPs	3–30	3	[42]
MnO_2_-nanozymes	0.16–36.93	0.005	[43]
MnO_2_ nanosheets	0.5–10	0.1	[44]
CQDs	0.05–20	0.016	[45]
CoFeCe-MOFs	7–70	6.8	[46]
2D Cu-TCPP(Fe) MOFs	0.1–60	0.0908	[47]
Por-ZnFe_2_O_4_/rGO	2–40	0.76	[48]

## Data Availability

The original contributions presented in this study are included in the article/supplementary material. Further inquiries can be directed to the corresponding authors.

## Supplementary Materials

The following supporting information can be downloaded at: https://www.mdpi.com/article/10.3390/biom15071002/s1, Table S1. Experimental Instruments and Manufacturers; Figure S1. Total spectrum of PCN-224-PDA-Pt; Figure S2. UV-Vis spectra of oxidized different chromogenic substrates; Figure S3. Hemolysis experiment diagram of PCN-224-PDA-Pt.
