# Supplementary material for: Platinum Nanoparticles Loaded in Polydopamine-Modified Porous Coordination Network-224 with Peroxidase-Like Activity for Sensitive Glutathione Detection
Source: Biomolecules. 2025 Jul 13;15(7):1002. doi: 10.3390/biom15071002 (PMC12293988; doi:10.3390/biom15071002)
Supplement: Supplementary file 1 [file biomolecules-15-01002-s001.zip › biomolecules-3709092-final-Supplementary Material.pdf]

SUPPORTING INFORMATION

Platinum nanoparticles loaded in polydopamine modified porous coordination network-224 with peroxidase-like activity for sensitive glutathione detection

Shoubei Gao [1,2], Mingyue Gao [2], Chenran Zhen [2], Yanshuai Cui [1]*, Xianbing Ji [1], Ruyu Li [2] and Longgang Wang [2]*

1. Department of Environmental Engineering, Hebei Key Laboratory of Agroecological Safety, Hebei University of Environmental Engineering, Qinhuangdao, 066102, China
2. State Key Laboratory of Metastable Materials Science and Technology, Hebei Key Laboratory of Nano-biotechnology, Hebei Key Laboratory of Applied Chemistry, Yanshan University, Qinhuangdao, 066004, China

* Corresponding author: cuiyanshuai@hebuee.edu.cn (Yanshuai Cui) lgwang@ysu.edu.cn (Longgang Wang)

Table S1 Experimental Instruments and Manufacturers

Instrument	Model	Manufacturer
UV-Vis Spectrophotometer	UV-TU1810	Beijing Purkinje General Instrument Co., Ltd.
Transmission Electron Microscope	HT 7700	Hitachi, Japan
X-ray Diffractometer	D-max-2500/PC	Rigaku, Japan
X-ray Photoelectron Spectrometer	Kratos AXIS Ultra	Thermo Fisher Scientific, USA
Freeze Dryer	FD-1B-50	Beijing Boyikang Lab Instruments
Multifunctional Microplate Reader	Spectra Max M2	Molecular Devices, USA
Thermogravimetric Analyzer	STA 409 PC	NETZSCH Instruments, Germany
Fourier Transform Infrared Spectrometer	Nicolet is10	Thermo Fisher Scientific, USA
Field Emission Scanning Electron Microscope	Zeiss SUPRA 55	Carl Zeiss, Germany
Fluorescence Spectrophotometer	F-7000	Hitachi, Japan

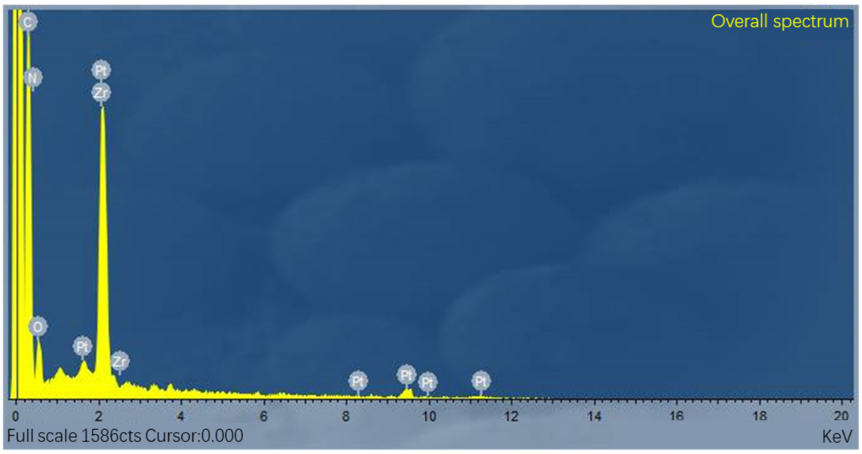

Figure S1 Total spectrum of PCN-224-PDA-Pt

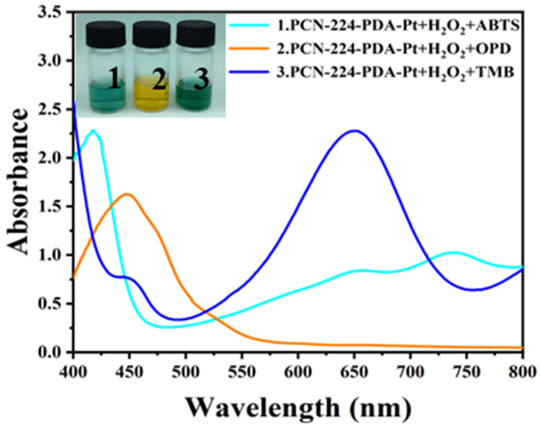

Figure S2 UV-Vis spectra of oxidized different chromogenic substrates

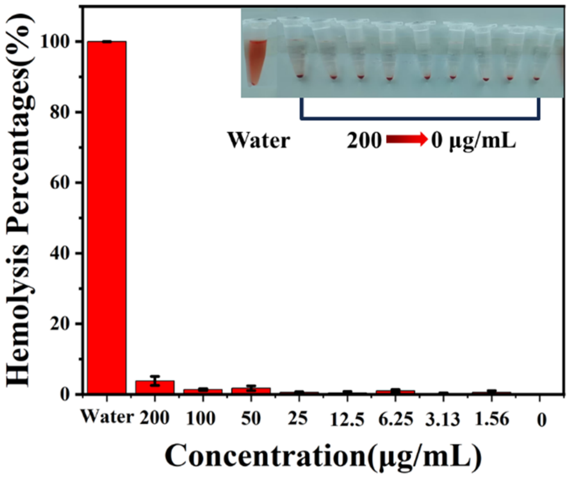

Figure S3 Hemolysis experiment diagram of PCN-224-PDA-Pt
